# Perceptions of, and Obstacles to, SARS-CoV-2 Vaccination Among Adults in Lebanon: Cross-sectional Online Survey

**DOI:** 10.2196/36827

**Published:** 2022-12-14

**Authors:** Nadeem Elias Abou-Arraj, Diana Maddah, Vanessa Buhamdan, Roua Abbas, Nadine Kamel Jawad, Fatima Karaki, Nael H Alami, Pascal Geldsetzer

**Affiliations:** 1 School of Public Health University of California Berkeley Berkeley, CA United States; 2 Department of Medicine Stanford University School of Medicine Stanford, CA United States; 3 Division of General Medicine Department of Medicine University of Michigan Medical School Ann Arbor, MI United States; 4 School of Health Sciences Modern University for Business and Science Beirut Lebanon; 5 Refugee and Asylum Seeker Health Initiative (RAHI) Department of Medicine University of California San Francisco San Francisco, CA United States; 6 Division of Primary Care and Population Health Department of Medicine Stanford University School of Medicine Stanford, CA United States; 7 Chan Zuckerberg Biohub San Francisco, CA United States

**Keywords:** Lebanon, COVID-19, SARS-CoV-2, coronavirus, vaccination, vaccine hesitancy, vaccine acceptance, health care system, misinformation, public health

## Abstract

**Background:**

The COVID-19 pandemic is an additional burden on Lebanon’s fragmented health care system and adds to its ongoing political, economic, and refugee crises. Vaccination is an important means of reducing the impact of the pandemic.

**Objective:**

Our study’s aims were to (1) assess the prevalences of intention to vaccinate and vaccine hesitancy in Lebanon; (2) determine how vaccine hesitancy in Lebanon varies by sociodemographic, economic, and geographic characteristics; and (3) understand individuals’ motivations for vaccinating as well as concerns and obstacles to vaccination.

**Methods:**

We performed a cross-sectional study from January 29, 2021, to March 11, 2021, using an online questionnaire of open- and closed-ended questions in Arabic via convenience “snowball” sampling to assess the perceptions of adults residing in Lebanon.

**Results:**

Of the 1185 adults who participated in the survey, 46.1% (95% CI: 43.2%-49.0%) intended to receive the SARS-CoV-2 vaccine when available to them, 19.0% (95% CI 16.8%-21.4%) indicated they would not, and 34.0% (95% CI 31.3%-36.8%) were unsure (with an additional 0.9% skipping this question). The most common reasons for hesitancy were concerns about safety, limited testing, side effects, and efficacy. Top motivations for vaccinating were to protect oneself, protect one’s family and the public, and end the pandemic. Despite financial hardships in Lebanon, barriers to vaccine access were not frequently described as concerns. Established health care facilities, rather than new temporary vaccination centers, were most frequently selected as preferred vaccination sites.

**Conclusions:**

Vaccine hesitancy appears to be high in Lebanon. Disseminating clear, consistent, evidence-based safety and efficacy information on vaccines may help reduce vaccine hesitancy, especially among the large proportion of adults who appear to be unsure about (rather than opposed to) vaccination.

## Introduction

As of November 9, 2021, Lebanon’s cumulative COVID-19 case count was 647,778 (95,695 cases per million), and at least 8556 people had died [1]. These numbers were likely underreported due to lack of testing and Lebanon’s fragmented health infrastructure [2]. In addition to the pandemic, Lebanon struggles with multiple challenges: a political crisis and economic collapse driven by corruption that began in 2019 and worsened throughout 2020 and 2021, leading to mistrust of the government, inflation, unemployment, poverty, and food insecurity, on top of the strain of being the country with the highest number of refugees per capita in the world due to the protracted Syrian refugee crisis [2-6]. Given Lebanon’s compounding crises and limited resources, mass vaccination is a challenging but vital mission. Understanding the population’s perceptions of SARS-CoV-2 vaccines is critical for implementing a successful vaccination campaign in the country, providing extra support for vulnerable populations, and bolstering demand for vaccination.

SARS-CoV-2 vaccination began in Lebanon on February 14, 2021 [7]. The Pfizer-BioNTech, Sputnik V, Oxford-AstraZeneca, and Sinopharm vaccines had been approved for use in Lebanon at the time [8]. Despite efforts from the Lebanese government and international aid to secure adequate supply of SARS-CoV-2 vaccines of multiple formulations, as of November 9, 2021, only 1,583,289 people had been fully vaccinated, representing approximately 23% of the total population [1,9-11]. Inadequate demand, possibly due to vaccine hesitancy and logistical challenges, appears to be part of the reason for slow vaccination; as of November 9, 2021, only roughly 43% of the population had registered with the online national vaccine registration tool through the Ministry of Public Health [7]. In December 2021, Lebanon implemented mandatory vaccination orders for civil servants and workers in education, tourism, public transportation, and health [12]. Given the sizeable unvaccinated population, understanding perceptions of the vaccines and perceived obstacles is important to increasing vaccination rates to end the pandemic.

An online survey of Lebanese in April 2020 and May 2020 found that 69% of the convenience sample (predominantly young, university-educated, and male) indicated they would receive a hypothetical vaccine against SARS-CoV-2, though at that time, none had been developed or approved [13]. Another online survey performed in Lebanon from November 2020 to December 2020, prior to administration of SARS-CoV-2 vaccines in Lebanon and across most of the world, found that only 21.4% of respondents wanted to receive the vaccine, but this study did not assess reasons for participants’ intentions [14]. These estimates of vaccine acceptance are in the lower end of the range of acceptance rates (13.3%-92%) found in surveys from countries in the Middle East and North Africa, which itself is a region with generally lower acceptance rates than other regions in the world [15].

Surveys assessing SARS-CoV-2 vaccine hesitancy around the world found that reasons for hesitancy include concerns about safety and side effects, doubts about efficacy, unfavorable personal risk and benefits assessments, and general mistrust in science and government [16-18]. Other barriers include distribution and uptake of the vaccines at scale, which are matters of logistics, health care access, and public perception of the vaccine [19,20]. These issues may be exacerbated in the Lebanese context given the deeply seated mistrust of government as well as compounding social crises. To our knowledge, no academic study has been done to elucidate the general public’s motivations behind intending to receive or not to receive SARS-CoV-2 vaccination since the vaccination campaign started in Lebanon.

Our study’s aims were to (1) assess rates of intention to vaccinate and vaccine hesitancy in Lebanon; (2) determine how vaccine hesitancy in Lebanon varies by sociodemographic, economic, and geographic characteristics; and (3) understand individuals’ motivations for vaccinating as well as concerns and obstacles to vaccination.

## Methods

### Study Design

We designed a cross-sectional descriptive study using an online Arabic survey. The survey was validated and revised in focus group piloting. We originally intended to distribute it to randomly selected Lebanese phone numbers to obtain an unbiased nationally representative sample, but during piloting, this method needed to be aborted because of low response rates (less than 1%), likely due to mistrust of messages received from an unknown phone number. Given these constraints, we changed our distribution methods to convenience “snowball” sampling, a method described in a later section that had successfully been used elsewhere in the Middle East [14,21]. The recruitment and survey period occurred from January 29, 2021, to March 11, 2021.

### Study Population

The target population was all adults living in Lebanon. Although all adults living in Lebanon were eligible, they needed to access the self-administered online survey tool on a mobile phone or computer. The survey was in Arabic, so participants had to be literate in Arabic or assisted by someone who was.

### Recruitment and Sampling:

Using a convenience “snowball” sampling method, the research team initiated recruitment by sending a recruitment message in Arabic to their contacts and organizations. Participants interested in the study proceeded to the survey via a link in the recruitment message, which also invited participants to forward the message to their contacts. It could be sent through WhatsApp, SMS, social media, and email. There was no follow-up to determine whether individuals who received the recruitment message completed or forwarded the survey. No incentives were provided for participation.

### Survey Tool and Data Collection

The survey (see Multimedia Appendix 1 and Multimedia Appendix 2) was created using the Qualtrics online survey platform [22]. It was anonymous, self-administered, and required basic literacy in Arabic. The survey content was created by the research team (the majority of whom were fluent in Arabic and English), used several other SARS-CoV-2 vaccine perception studies as a guide [23,24], and was tailored to the Lebanese context. It was piloted in small groups of contacts of the research team of varying educational backgrounds and health literacy and revised prior to survey launch.

The survey included 31 multiple-choice and free-response questions (depending on branch points, participants were not asked each question), divided into an introduction with the informed consent document, followed by questions about screening, demographics, experience with COVID-19, and perceptions of SARS-CoV-2 vaccination. No identifying data were collected. Participants were asked to provide informed consent and affirm that they were 18 years or older, were living in Lebanon, and had heard of “coronavirus” (as SARS-CoV-2 and COVID-19 are referred to in Lebanon). If they declined, the survey automatically ended. If they passed these questions, they proceeded with the survey. After starting the survey, participants had 48 hours before responses were automatically recorded. Participants were prevented from multiple survey attempts on the same device via a Qualtrics feature based on browser cookies.

### Ethical Approval and Informed Consent

This study was approved by the Institutional Review Boards of the University of California, Berkeley (Protocol number 2020-11-13811); the Modern University for Business and Science, Lebanon (Project number MU-20201207-19); and Stanford University (by reliance agreement with the University of California, Berkeley, eProtocol number 59832). Informed consent was obtained from all participants prior to the survey; a waiver of written informed consent was granted given that written consent would have been the only identifying information collected.

### Data Analysis

Data were downloaded as a .csv file and translated into English using Microsoft Excel [25]. Data cleaning and statistical analysis were performed in R computer software and focused on description rather than identifying causal links [26].

#### Quality Measures

Several quality measures were implemented. To ensure that participants had baseline familiarity with the survey topics, a screening question asked whether participants had heard of “coronavirus”; only 5 participants (5/1185, 0.42%) were excluded because of not being aware of “coronavirus.” To identify participants who randomly clicked through the survey, a filter was applied to detect participants who completed the survey in less than 120 seconds; no participants did so.

#### Quantitative Data Analysis

For some multiple-choice questions, similar categorical responses were consolidated into binary or fewer categories to facilitate interpretation. For binary and categorical variables, the absolute number and relative proportions of participants who selected each response were calculated. Wilson score 95% confidence intervals were calculated for proportions.

Given that the survey period spanned the initiation of SARS-CoV-2 vaccination in Lebanon, a subanalysis was performed in which participants were divided by whether they completed the survey before or after vaccine initiation. For each of these subgroups, sample demographics were recalculated. We did not use sampling weights in our analysis given that this was a nonprobabilistic sample of the Lebanese population that was unlikely to be representative of the general population even after weighting.

#### Qualitative Data Analysis

If participants indicated that they intended to vaccinate, they were then asked to explain why they intended, did not intend, or were uncertain about vaccinating. These open-ended responses were thematically, iteratively coded using a uniform protocol to facilitate analysis (see Multimedia Appendix 3).

## Results

### Sample Characteristics

Of the 1390 participants who initiated the survey, 1185 (85.3%) provided informed consent and passed screening to begin the main survey. Among this group, 1103 (1103/1185, 93.1%) participants completed the entire survey. Selected sample demographic characteristics of those who passed screening are summarized in Table 1. For full sample demographic characteristics, see the expanded table in Table S1 in Multimedia Appendix 4.

Compared with the demographics of residents of Lebanon in general, our sample population had a higher representation of individuals who identified with the Druze religion and were young, female, well-educated, and from the Mount Lebanon region (Table 1). Underrepresented in our sample were older adults; refugees; non-Lebanese citizens; members of the Sunni and Shi’a religions; and individuals from the less populated governorates of Akkar, Baalbek-Hermel, Nabatieh, North, and South.

**Table 1:** Participant characteristics (n=1185).

Characteristic^a^		All participants, n (%)	Round 1 participants^b^ (n=840), n (%)	Round 2 participants^c^ (n=345), n (%)	Estimates for the Lebanese population^d^ [6,27-30], %
**Gender**					
	Female	685 (62.1)	479 (60.1)	206 (65.6)	51.6
	Male	388 (35.2)	285 (36.1)	103 (32.8)	48.4
	Other	7 (0.1)	6 (0.8)	1 (0.3)	N/A^e^
	Skip this question	23 (2.1)	19 (2.4)	4 (1.3)	—^f^
**Age (years)**					
	18-34	745 (62.8)	493 (58.7)	252 (73.1)	32.0^g^
	35-54	318 (26.8)	248 (29.5)	70 (20.3)	23.5
	≥55	122 (10.3)	99 (11.8)	23 (6.7)	19.8
**Governorate**					
	Beirut	111 (10.1)	89 (11.3)	22 (7.0)	7.1
	Mount Lebanon	633 (57.4)	559 (70.8)	74 (23.6)	42.3
	Other	342 (31.0)	125 (15.9)	217 (69.1)	50.6
	Skip this question	17 (1.5)	16 (2.0)	1 (0.3)	—
**Religion**					
	Christian	242 (21.9)	199 (25.2)	43 (13.7)	32.4
	Druze	355 (32.2)	308 (39.0)	47 (15.0)	4.5
	Shi'a	136 (12.3)	46 (5.8)	90 (28.7)	31.0
	Sunni	124 (11.2)	64 (8.1)	60 (19.1)	31.9
	No religion	65 (5.9)	48 (6.1)	17 (5.4)	N/A
	Other	6 (0.5)	3 (0.4)	3 (1.0)	0.3
	Skip this question	175 (15.9)	121 (15.3)	54 (17.2)	—
**Highest education level**					
	Completed high school, technical school, or less	200 (18.1)	143 (18.1)	57 (18.2)	78.6
	Completed some college or more	891 (80.9)	637 (80.8)	254 (80.9)	21.4
	Skip this question	11 (1.0)	8 (1.0)	3 (1.0)	—
**Employment status**					
	Employed	586 (51.5)	412 (52.2)	156 (49.7)	N/A
	Student	179 (16.2)	117 (14.8)	62 (19.7)	N/A
	Unemployed, not seeking work	145 (13.1)	122 (15.5)	23 (7.3)	N/A
	Unemployed, seeking work	154 (14.0)	101 (12.8)	53 (16.9)	33.0^h^
	Skip this question	57 (5.2)	37 (4.7)	20 (6.3)	—
**Citizenship^i^**					
	Lebanon	1038 (94.1)	757 (96.0)	281 (89.5)	79.8
	Not a citizen of Lebanon	53 (4.8)	24 (3.0)	25 (8.0)	20.2
	Skip this question	12 (1.1)	8 (1.0)	8 (2.5)	—
**Refugee**					
	Yes	56 (5.1)	29 (3.7)	27 (8.6)	21.9^h^
	No	1015 (92.0)	737 (93.4)	278 (88.5)	78.1^h^
	Skip this question	32 (2.9)	23 (2.9)	9 (2.9)	—

^a^Because participants were not forced to answer all questions, each question has a different denominator.

^b^Those who completed the survey in the first round, prior to initiation of vaccination in Lebanon on February 13, 2021.

^c^Those who completed the survey in the second round, after initiation of vaccination in Lebanon.

^d^Unless otherwise noted, estimates were obtained from government source that excluded refugees and used 4.84 million (2018) as total population.

^e^N/A: not available in the data source.

^f^Not applicable.

^g^15-34 years old.

^h^Estimates were obtained from a source that included refugees and used 6.86 million (2020) as the total population.

^i^Participants could select multiple answers, so proportions were calculated using a denominator of all participants who selected an answer for the question.

### Quantitative Analysis

#### Intentions to Vaccinate

We found that 46.1% (95% CI 43.2%-49.0%) of our survey participants intended to receive the SARS-CoV-2 vaccine when available, 19.0% (95% CI 16.8%-21.4%) indicated they would not get vaccinated, and 34.0% (95% CI 31.3%-36.8%) were unsure about vaccination (with an additional 0.9% skipping this question; Table 2).

**Table 2:** Intentions about vaccination by sociodemographic characteristics and experience with COVID-19 for everyone who answered the intention to vaccinate question (n=1185).

Characteristic^a^			Entire sample, n (%)		Intend to vaccinate, % (95% CI)		Do not intend to vaccinate, % (95% CI)		Unsure about vaccine, % (95% CI)
All participants			—^b^		46.1 (43.2-49.0)		19.0 (16.8-21.4)		34.0 (31.3-6.8)
**Gender**									
	Female	682 (63.6)		42.8 (39.1-46.6)		21.1 (18.1-24.4)		36.1 (32.5-39.8)	
	Male	385 (35.9)		55.6 (50.5-60.6)		15.6 (12.2-19.7)		28.8 (24.4-33.7)	
	Other	6 (0.6)		33.3 (6.0-75.9)		50.0 (18.8-81.2)		16.7 (0.9-63.5)	
**Age (years)**									
	18-24	364 (31.0)		39.6 (34.5-44.8)		22.5 (18.4-27.2)		37.9 (32.9-43.1)	
	25-34	372 (31.7)		45.4 (40.3-50.6)		23.1 (19.0-27.8)		31.5 (26.8-36.5)	
	35-44	191 (16.3)		50.8 (43.5-58.0)		14.1 (9.7-20.0)		35.1 (28.4-42.3)	
	45-54	125 (10.6)		51.2 (42.1-60.2)		12.0 (7.1-19.3)		36.8 (28.5-45.9)	
	55-64	88 (7.5)		60.2 (49.2-70.3)		12.5 (6.7-21.7)		27.3 (18.6-38.0)	
	≥65	34 (2.9)		55.9 (38.1-72.4)		11.8 (3.8-28.4)		32.4 (18.0-50.6)	
**Governorate**									
	Baalbek-Hermel	20 (1.9)		50.0 (29.9-70.1)		15.0 (4.0-38.9)		35.0 (16.3-59.1)	
	Beqaa	98 (9.1)		59.2 (48.8-68.9)		12.2 (6.8-20.8)		28.6 (20.1-38.7)	
	Beirut	111 (10.3)		53.2 (43.5-62.6)		16.2 (10.1-24.7)		30.6 (22.4-40.2)	
	Mount Lebanon	632 (58.6)		42.2 (38.4-46.2)		22.8 (19.6-26.3)		35.0 (31.3-38.8)	
	South	70 (6.5)		57.1 (44.8-68.7)		10.0 (4.5-20.1)		32.9 (22.4-45.2)	
	Akkar	13 (1.2)		84.6 (53.7-97.3)		7.7 (0.4-37.9)		7.7 (0.4-37.9)	
	North	58 (5.4)		46.6 (33.5-60.0)		17.2 (9.0-29.9)		36.2 (24.3-49.9)	
	Nabatieh	77 (7.1)		50.6 (39.1-62.1)		18.2 (10.6-29.0)		31.2 (21.4-42.9)	
**Religion**									
	Christian	242 (22.1)		60.3 (53.8-66.4)		9.5 (6.2-14.1)		30.2 (24.5-36.4)	
	Druze	354 (32.3)		35.6 (30.6-40.9)		25.4 (21.0-30.3)		39.0 (33.9-44.3)	
	Shi'a	134 (12.2)		48.5 (39.8-57.3)		16.4 (10.8-24.0)		35.1 (27.2-43.8)	
	Sunni	122 (11.1)		57.4 (48.1-66.2)		21.3 (14.6-29.8)		21.3 (14.6-29.8)	
	No religion	65 (5.9)		58.5 (45.6-70.3)		16.9 (9.1-28.7)		24.6 (15.1-37.1)	
	Other	5 (0.5)		20.0 (1.1-70.1)		0.0 (0.0-53.7)		80.0 (29.9-99.0)	
	Skip this question	173 (15.8)		39.9 (32.6-47.6)		24.9 (18.8-32.1)		35.3 (28.3-42.9)	
**Education**									
	Completed high school, technical school, or less	197 (18.2)		42.1 (35.2-49.4)		21.3 (16.0-27.8)		36.5 (30.0-43.7)	
	Completed some college or more	886 (81.8)		48.8 (45.4-52.1)		18.5 (16.0-21.3)		32.7 (29.7-35.9)	
**Employment**									
	Employed	566 (54.3)		51.6 (47.4-55.8)		17.1 (14.2-20.6)		31.3 (27.5-35.3)	
	Student	179 (17.2)		43.0 (35.7-50.6)		21.2 (15.6-28.1)		35.8 (28.8-43.3)	
	Unemployed, not seeking work	145 (13.9)		42.1 (34.0-50.6)		18.6 (12.8-26.1)		39.3 (31.4-47.8)	
	Unemployed, seeking work	152 (14.6)		41.4 (33.6-49.7)		23.0 (16.8-30.7)		35.5 (28.1-30.7)	
**Annual income (LL^c^)**									
	<1,000,000	121 (11.1)		34.7 (26.4-44.0)		25.6 (18.3-34.5)		39.7 (31.0-49.0)	
	1,000,000-9,999,999	287 (26.2)		45.6 (39.8-51.6)		18.8 (14.6-23.9)		35.5 (30.1-41.4)	
	10,000,000-19,999,999	99 (9.0)		47.5 (37.4-57.7)		24.2 (16.4-34.1)		28.3 (19.9-38.4)	
	20,000,000-69,999,999	131 (12.0)		63.4 (54.4-71.5)		9.2 (5.0-15.8)		27.5 (20.2-36.1)	
	≥70,000,000	61 (5.6)		68.8 (55.6-79.8)		6.6 (2.1-16.7)		24.6 (14.8-37.6)	
	Skip this question	396 (36.2)		42.9 (38.0-48.0)		22.7 (18.8-27.2)		34.3 (29.7-39.3)	
**Citizenship^d^**									
	Lebanon	1033 (94.7)		47.2 (44.2-50.3)		19.1 (16.7-21.6)		33.7 (30.8-36.7)	
	Syria	27 (2.5)		40.7 (23.0-61.0)		37.0 (20.1-57.5)		22.2 (9.4-42.7)	
	Palestine	17 (1.6)		58.8 (33.5-80.6)		11.8 (2.1-37.7)		29.4 (11.4-56.0)	
	European or North American country	40 (3.7)		62.5 (45.8-76.8)		12.5 (4.7-27.6)		25.0 (13.2-41.5)	
	Other country	22 (2.0)		31.8 (14.7-54.8)		18.2 (6.0-41.0)		50.0 (30.7-69.3)	
	Multiple countries	53 (4.9)		54.7 (40.6-68.2)		13.2 (5.9-26.0)		32.1 (20.3-46.4)	
**Refugee**									
	Yes	54 (5.1)		50.0 (37.1-62.9)		25.9 (15.4-39.9)		24.1 (13.9-37.9)	
	No	1009 (94.9)		46.9 (43.8-50.0)		19.0 (16.7-21.6)		34.1 (31.2-37.1)	
**Completed survey after initiation of vaccination in Lebanon**									
	Yes	339 (28.9)		56.9 (51.5-62.2)		12.7 (9.4-16.8)		30.4 (25.6-35.6)	
	No	835 (71.1)		42.3 (38.9-45.7)		21.8 (19.1-24.8)		35.9 (32.7-39.3)	
**Correct knowledge of transmission^e^**									
	Yes	1076 (99.1)		48.0 (45.1-51.1)		18.1 (15.9-20.6)		33.8 (31.0-36.8)	
	No	10 (0.9)		50.0 (23.7-76.3)		40.0 (13.7-72.6)		10.0 (0.5-45.9)	
**Personal history of COVID-19**									
	Yes	321 (28.9)		42.9 (37.5-48.6)		21.6 (17.3-26.6)		35.4 (30.2-41.0)	
	No	790 (71.1)		48.8 (45.2-52.3)		18.5 (15.9-21.5)		32.7 (29.4-36.1)	
**Close acquaintance with history of COVID-19**									
	Yes	1056 (94.2)		48.0 (44.9-51.0)		18.8 (16.5-21.3)		33.3 (30.4-36.2)	
	No	65 (5.8)		33.3 (22.2-46.4)		30.2 (19.6-43.2)		36.5 (25.0-49.6)	
**Mask wearing outside home**									
	Always or most of the time	1011 (91.2)		50.0 (46.8-53.1)		16.3 (14.1-18.8)		33.7 (30.8-36.8)	
	Sometimes, rarely, or never	97 (8.8)		18.6 (11.7-28.0)		48.5 (38.3-58.8)		33.0 (24.0-43.4)	
**Top 3 news sources^d^**									
	Printed newspaper or magazine	86 (7.8)		64.0 (52.8-73.8)		9.3 (4.4-18.0)		26.7 (18.0-37.6)	
	Radio	33 (3.0)		69.7 (51.1-83.8)		15.2 (5.7-32.7)		15.2 (5.7-32.7)	
	Television	718 (65.1)		47.2 (43.5-50.9)		17.1 (14.5-20.1)		35.7 (32.2-39.3)	
	Social media (eg, Facebook, Twitter, YouTube, WhatsApp)	670 (60.1)		43.7 (39.9-47.6)		19.4 (16.5-22.6)		36.9 (33.2-40.7)	
	Internet but not social media (eg, websites)	609 (55.2)		53.5 (49.5-57.5)		17.4 (14.5-20.7)		29.1 (25.5-32.9)	
	Talking to friends or family	282 (25.6]		37.6 (32.0-43.6)		24.1 (19.3-29.6)		38.3 (32.6-44.3)	
	Religious leaders	6 (0.5)		16.7 (0.9-63.5)		83.3 (36.5-99.1)		0.0 (0.0-48.3)	

^a^For the analysis of each characteristic, we omitted participants who skipped the question, unless >10% of participants for that question skipped the question, in which case those who skipped the characteristic question were included in the analysis. We then calculated the proportion of each characteristic subcategory by intention to vaccinate, calculating Wilson score CIs.

^b^Not applicable.

^c^LL: Lebanese Lira.

^d^The survey allowed participants to choose multiple answers for this characteristic; consequently, the sum of all subcategories does not equal the number of all participants who answered the question.

^e^“Correct Knowledge” indicated a correct response to a multiple-choice question asking, “In your understanding, how does someone become infected with coronavirus? Choose the best single answer.” The correct response was “Being physically close to an infected person.” Incorrect responses were “Eating raw food or untreated water” and “Being bitten by an insect.”

#### Intentions to Vaccinate by Demographic Characteristics

**Figure 1:**
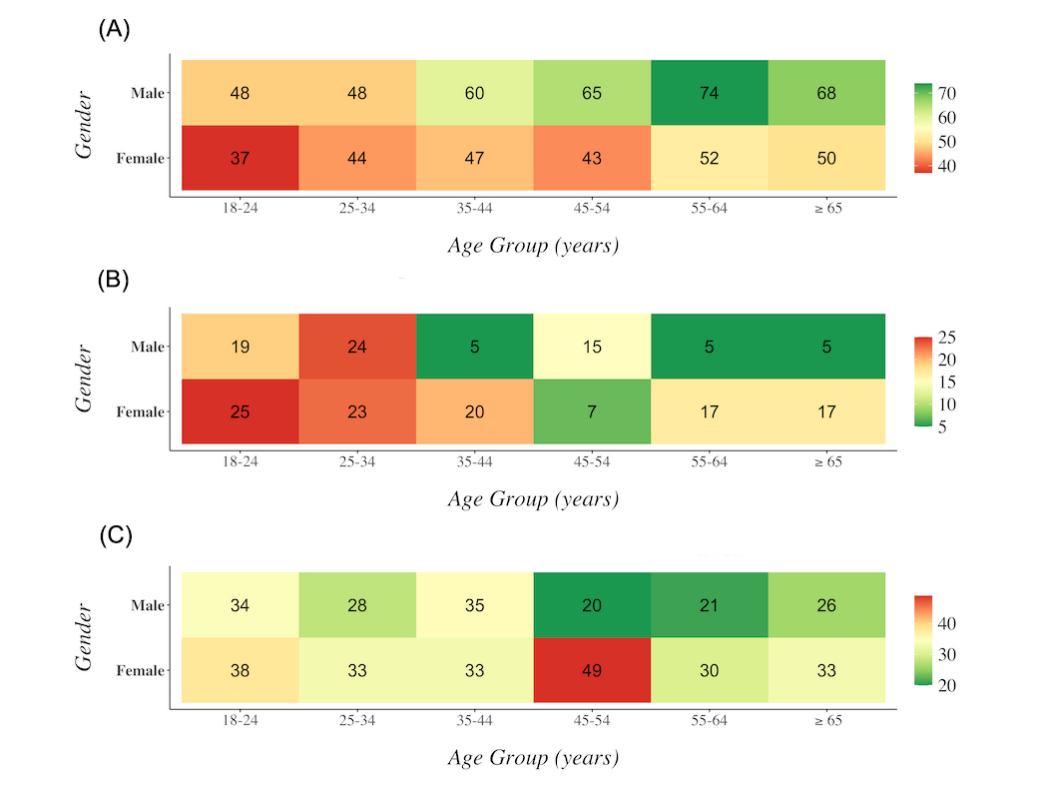
Intention to receive SARS-CoV-2 vaccine by age group and gender for those who provided age, gender, and their intentions about vaccination, reported as the percentage who (A) intended to receive the SARS-CoV-2 vaccine, (B) did not intend to receive the SARS-CoV-2 vaccine, (C) were not sure about receiving the SARS-CoV-2 vaccine. As only 7 participants identified as “other” gender, only participants identifying as “male” or “female” were included.

Participants were more likely to intend to vaccinate if they identified as male; lived in the Beqaa governorate (Mount Lebanon as reference); were Christian, Sunni, or affiliated with no religion (Druze as reference); and had a higher household income (Table 2 and Figure 1).

There were less strong but still apparent trends toward higher proportions of participants’ intending to vaccinate if they were older, lived in Beirut or Akkar governorates (Mount Lebanon as reference), had attained higher educational status, or were employed. There were no apparent differences nor trends in intention to vaccinate by citizenship or whether participants identified as refugees.

#### Intentions to Vaccinate Before and After Initiation of Vaccination in Lebanon

Those who completed the survey after initiation of vaccination were more likely to intend to vaccinate (56.9%, 95% CI 51.5%-62.2%) when compared with those who completed the survey before initiation of vaccination (42.3%, 95% CI 38.9%-45.7%; Table 2). Importantly, these groups were contacted differently and differed in several key demographic characteristics: The group of participants who responded after initiation of vaccination was younger and had a higher proportion of participants who lived outside of the Mount Lebanon region, identified as Shi’a or Sunni rather than Druze or Christian, had European or North American or multiple citizenships, and identified as refugees (Table 1).

#### Logistical Considerations About Vaccination

**Figure 2:**
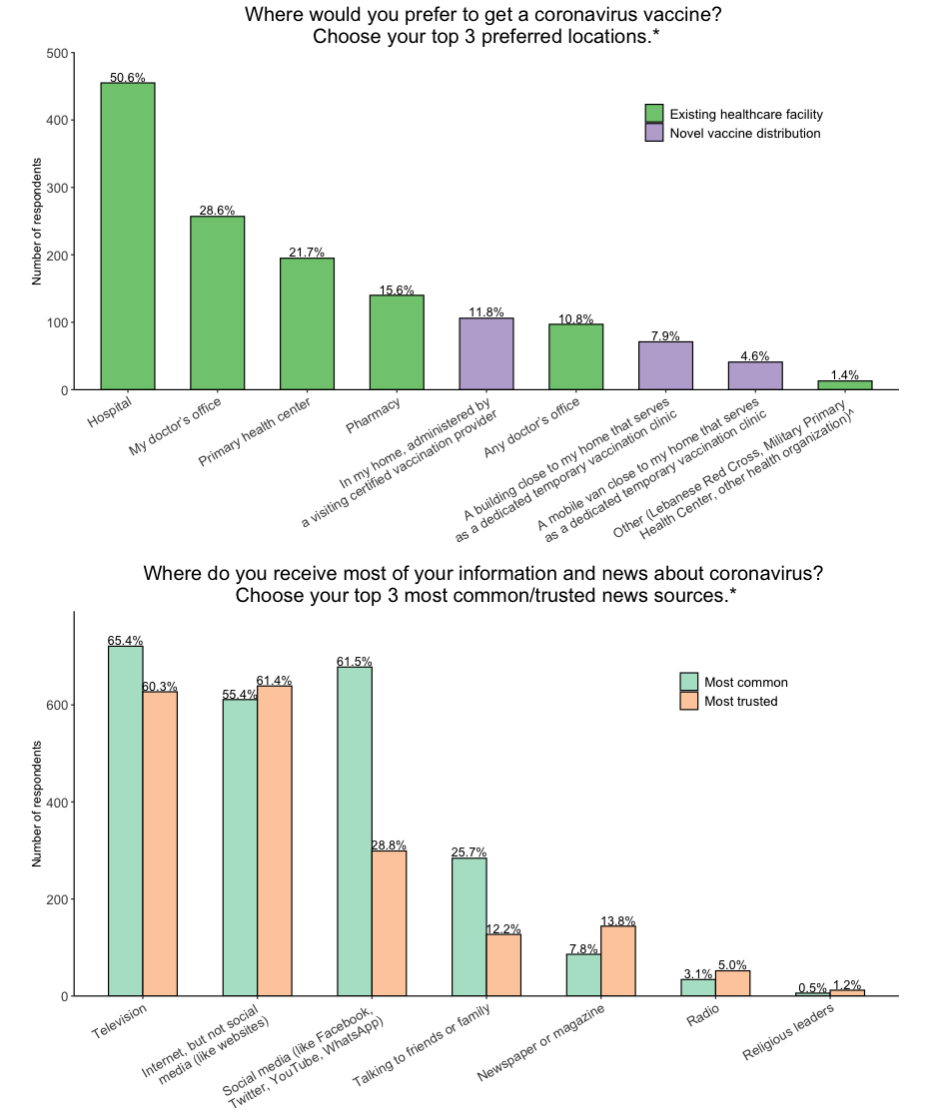
Top 3 ranked responses from each participant for the logistical considerations of where participants would prefer to get a coronavirus vaccine and which news sources were the most commonly used or trusted for coronavirus information. If participants selected “Other,” they could write in their own response. The 3 responses in parentheses (Lebanese Red Cross, Military Primary Health Center, other health organization) encompass the written-in responses.

The most commonly selected preferred locations to get vaccinated were hospitals, doctors’ offices, primary health centers, and pharmacies (Figure 2, Multimedia Appendix 5). Temporary vaccination sites were not popular. The most common sources of news about coronavirus were television, social media, and other internet websites (Figure 2, Multimedia Appendix 5). Very few participants (6/1185, 0.5%) reported commonly relying on religious leaders for coronavirus news (Multimedia Appendix 5). Participants trusted television and internet websites more than social media (Multimedia Appendix 5). Among participants who did not intend to vaccinate or were uncertain about vaccination, less than 2% (10/599, 1.7%) stated that a monetary incentive would persuade them to become vaccinated (Multimedia Appendix 5).

### Qualitative Analysis: Motivations for and Concerns About Vaccination

Most frequently, participants intending to vaccinate cited the following motivations: to protect themselves, their families, and the public and to end the pandemic and return to normal life (Table 3). Somewhat frequently given reasons for intending to vaccinate included trusting science and research and feeling like there was “no other choice” given the state of the pandemic.

**Table 3:** Summary of coded open-ended responses describing motivations for intentions about vaccination.

Motivation	Most frequent codes (descending frequency)^a^	Less frequent codes (descending frequency)^b^
**Motivations for intending to vaccinate**		
	To protect myself To protect and limit spread among the public To end the pandemic and return to normalcy To protect my family	I do not have another better choice or solution. I trust it based on science and research. I want to help achieve “herd immunity.“ I have other medical conditions that make my risk of illness higher. My job puts me at risk of contracting COVID-19.
**Motivations for not intending to vaccinate**		
	I am concerned about safety and limited testing. I am concerned about potential side effects. Nonspecific mistrust of the SARS-CoV-2 vaccines I am worried that the vaccines are not effective.	I do not trust the Lebanese government. I do not trust Lebanese health care or distribution systems. COVID-19 and vaccines are a scam or conspiracy to make money. General vaccine hesitancy; I am against all vaccines. Problems or concerns with ingredients I do not need the vaccine because I already had COVID-19. COVID-19 is not a threat to me or in general. Risks of vaccination are not worth the potential benefits. Society and science in general do not know enough about COVID-19 (causes, symptoms, effects).
**Motivations for uncertainty about vaccination**		
	I am concerned about safety and limited testing. I am concerned about potential side effects. I am worried that the vaccines are not effective.	I do not think I need the vaccine because I already had COVID-19. I do not trust the Lebanese government. Nonspecific mistrust of the SARS-CoV-2 vaccines I do not have enough information about the vaccines. I want more time or more information before I decide which vaccine to take. I have received conflicting information about the vaccine from one or more sources. I have concerns about mRNA technology. I do not trust Lebanese health care or distribution systems. I think the vaccines in Lebanon will not be the true coronavirus vaccine or that they will be tampered with.

^a^“Most frequent codes” were observed in ≥15% of responses to the question.

^b^“Less frequent codes” were observed in 3%-15% of responses to the question.

Among participants who did not intend to be vaccinated, the most frequent reasons for vaccine hesitancy were concerns about safety given the fast development and limited testing of the vaccines, fears about side effects, and doubts about efficacy (Table 3). Somewhat frequently cited concerns leading to vaccine hesitancy were mistrust in the Lebanese government.

The most commonly provided reasons for uncertainty about whether participants planned to receive the vaccine were similar: concerns about safety given the fast development and limited testing of the vaccines, fears about side effects, and doubts about efficacy (Table 3). Somewhat frequently mentioned concerns included mistrust of the Lebanese government and health care system, potential fraud in storage or marketing of the vaccines, wanting more information about the vaccines, and needing more time to decide which vaccine to receive.

## Discussion

### Principal Findings

Our findings of rates of vaccine acceptance and vaccine hesitancy in Lebanon were similar to those of studies in other countries in the Middle East and across the world. The vaccine acceptance of 46.1% in our sample was similar to rates in Kuwait (53.1%) and Qatar (45%-60%), higher than rates in Jordan (28.4%), and somewhat lower than in Saudi Arabia (64.7%) [21,31-33]. Rates of vaccine acceptance in our study were also similar to a large survey of 15 developed countries across the globe, in which 54% of respondents indicated they intend to vaccinate [34]. However, as systematic reviews of vaccine perception studies showed, vaccine acceptance and hesitancy have varied with time throughout the pandemic, with a trend toward decreasing acceptance throughout 2020 [18,35]. Indeed, our results show lower rates of vaccine acceptance than a survey in April 2020 and May 2020 that included a question about acceptance of hypothetical SARS-CoV-2 vaccines, in which 69.3% of Lebanese residents stated they would be willing to receive a vaccine; it is important to note that, at this time, no vaccines were developed or approved and the survey’s population was predominantly male and younger than ours [13]. In contrast, our study found higher rates of intent to vaccinate than an online convenience survey in November 2020 and December 2020, in which only 21.4% of participants intended to be vaccinated [14]. Notably, the study’s sample was smaller, more predominantly female, and younger than ours, and it took place prior to mass vaccination in most of the world, including in Lebanon. It also did not directly assess motivations for intention to or not to vaccinate. Our study found lower rates of vaccine acceptance than the vaccine acceptance rate of 87% among 800 university students surveyed in May 2021 and June 2021 at a prominent Lebanese university [36].

Our findings of trends toward increased vaccine hesitancy in women, younger age groups, unemployed individuals, and individuals with lower education attainment are generally consistent with findings in the Middle East and globally, with the exception that, in Kuwait and Qatar, hesitancy was higher in older populations [14,31,32,35,37-39]. Although we attempted to assess the association of vaccine hesitancy with religion and income, 2 important demographic variables in Lebanon, 15.9% and 36.3% of participants skipped questions about religion and income, respectively, demonstrating the topics’ sensitive natures. Therefore, we do not recommend inferences about differences in vaccine acceptance or hesitancy by religion or income from our study. Similarly, refugees (56/1185, 5.1%) were underrepresented in our sample. Although no significant difference in vaccine acceptance by refugee status emerged, further focus on vaccination in refugees in Lebanon is merited given their multiple vulnerabilities. One survey among Syrian refugees in Lebanon in January 2021 and February 2021 found that 66% of respondents would accept a “safe and free” vaccine [40].

The timing of our survey period spanned the initiation of vaccination in Lebanon. Although our data suggested increased vaccine acceptance among participants who completed the survey after initiation of vaccination, this must be interpreted cautiously. We believe that the differences in vaccine acceptance before and after initiation of vaccination more likely reflect significant differences in populations surveyed during these periods, given the selection bias inherent in the convenience sampling method.

The logistical considerations about which we asked can provide some guidance for vaccination efforts in Lebanon. The most commonly selected sources of news about COVID-19 were television, social media, and internet websites, with television and internet websites most trusted. Focusing dissemination of vaccine promotion efforts on television, social media, and internet websites could be most efficient to reach those with vaccine hesitancy.

Survey respondents reported preferring to receive the vaccine at familiar, established health care sites. Although temporary dedicated vaccination centers were not popular, a small but significant number of participants (106/899, 11.8%) stated they would prefer vaccination at home by a visiting medical professional, a potentially important means of reaching vulnerable patients unable to travel. We also asked whether a financial incentive would change participants’ minds so that they decide to vaccinate; overwhelmingly, they indicated that it would not (589/599, 98.3%).

Despite financial hardships in Lebanon, barriers to vaccine access (cost, transportation, proximity to medical care) were not cited frequently as concerns in our study. This might be explained by the fact that it has become common for governments worldwide to distribute the vaccine free of charge. There were relatively few concerns about vaccine properties like number of required doses, country of origin, or specific vaccine brands. Also uncommon was opposition to vaccines in general (ie, not specific to SARS-CoV-2 vaccines). This is consistent with previous studies in Lebanon showing moderate uptake of routine vaccinations [41-45]. Although several participants in our study cited conspiracy theories as reasons for not vaccinating, these were relatively uncommon, especially compared with a study in other Arabic-speaking countries, which found rates of belief in conspiracy theories of over 50% [33].

Perhaps the most actionable information generated in this study involves motivations for vaccine acceptance and vaccine hesitancy. The relatively large proportion of participants who were uncertain about vaccination—34.0%—provides a public health opportunity and imperative in the effort to achieve mass vaccination in Lebanon. The majority of concerns about SARS-CoV-2 vaccination involved absence of reliable information and data regarding safety, testing, and efficacy. Increasing public availability in Lebanon of high-quality, accessible information about the SARS-CoV-2 vaccines could assuage such concerns and increase vaccine acceptance. Vaccine promotion campaigns could also use messaging based on the most frequently provided reasons for intending to vaccinate: to protect oneself, protect loved ones, and end the pandemic. Based on our results about common COVID-19 news sources, we recommend disseminating clear, consistent, verifiable safety and efficacy information on television, social media, and news websites. Given the prevalence of mistrust in the government, third parties (like health care organizations) might be most trusted, especially if they are able to provide transparency and reassurance about proper vaccine acquisition and storage to allay concerns about fraud.

### Limitations

Our study has several important limitations. First and foremost, our sample is unlikely to be representative of the general population because of the sampling strategy. Although we attempted to mitigate this by collecting important demographic and experiential characteristics, the bias remains, and several important demographic groups were underrepresented, most notably older adults, members of Shi’a and Sunni religions, residents outside of Beirut and Mount Lebanon, non-Lebanese citizens, and those who identify as refugees. Second, participation required literacy in Arabic, internet access, and digital literacy, potentially excluding some populations (though >80% of refugees in Lebanon have access to mobile technology like WhatsApp) [46]. Third, some participants did not answer all questions, possibly causing nonresponse bias. Finally, vaccine hesitancy, perceptions, and concerns may be changing rapidly over time; our results should be interpreted as pertaining to the time period during which the survey was conducted.

### Conclusions

This cross-sectional study assessed intentions to vaccinate against SARS-CoV-2 among adults residing in Lebanon, analyzed characteristics associated with vaccine acceptance and hesitancy, and described motivations for and concerns about vaccination. We recommend disseminating clear, consistent, evidence-based safety and efficacy information on vaccines via most commonly selected news sources: television, social media, and news websites. Repeated assessments of intentions to vaccinate, concerns regarding vaccination, and changes in motivations should be performed, especially with the goal of assessing the perspectives and needs of populations that were underrepresented in this study.

## Appendix

Multimedia Appendix 1 Survey tool in English.

Multimedia Appendix 2 Survey tool in Arabic.

Multimedia Appendix 3 Coding protocol for free-response questions assessing motivations for intent to or not to vaccinate.

Multimedia Appendix 4 Table 1S: Unabridged characteristics of all the participants who passed screening.

Multimedia Appendix 5 Table 2S: Additional logistical considerations about vaccination.
