# Supplementary material for: Perceptions of, and Obstacles to, SARS-CoV-2 Vaccination Among Adults in Lebanon: Cross-sectional Online Survey
Source: JMIR Form Res. 2022 Dec 14;6(12):e36827. doi: 10.2196/36827 (PMC9762140; doi:10.2196/36827)
Supplement: Multimedia Appendix 4 [file formative_v6i12e36827_app4.docx]

**Table S1**. Unabridged characteristics of all the participants who passed screening.

| **Characteristic** | **All participants, n (%)** | **Round 1 participants, n (%)** | **Round 2 participants, n (%)** | **Estimates for Lebanese population^b^** [6,27–30] |
| --- | --- | --- | --- | --- |
| **Total participants** | 1185 (100) | 840 (70.9) | 345 (29.1) |  |
| **Gender** |  |  |  |  |
| Female | 685 (62.1) | 479 (60.1) | 206 (65.6) | 51.6% |
| Male | 388 (35.2) | 285 (36.1) | 103 (32.8) | 48.4% |
| Other | 7 (0.1) | 6 (0.8) | 1 (0.3) | NA |
| Skip this question | 23 (2.1) | 19 (2.4) | 4 (1.3) |  |
| **Age** |  |  |  |  |
| 18-24 years old | 369 (31.1) | 246 (29.3) | 123 (35.7) | 15-24 years old: 17.5% |
| 25-34 years old | 376 (31.7) | 247 (29.4) | 129 (37.4) | 14.5% |
| 35-44 years old | 192 (16.2) | 144 (17.1) | 48 (13.9) | 11.8% |
| 45-54 years old | 126 (10.6) | 104 (12.4) | 22 (6.4) | 11.7% |
| 55-64 years old | 88 (7.4) | 69 (8.2) | 19 (5.5) | 9.6% |
| 65 years old or older | 34 (2.9) | 30 (3.6) | 4 (1.2) | 10.2% |
| **Governorate** |  |  |  |  |
| Baalbek-Hermel | 21 (1.9) | 6 (0.8) | 15 (4.8) | 5.1% |
| Beqaa | 100 (9.1) | 47 (6.0) | 53 (16.9) | 6.2% |
| Beirut | 111 (10.1) | 89 (11.3) | 22 (7.0) | 7.1% |
| Mount Lebanon | 633 (57.4) | 559 (70.8) | 74 (23.6) | 42.3% |
| South | 72 (6.5) | 26 (3.3) | 46 (14.6) | 12.2% |
| Akkar | 13 (1.2) | 3 (0.4) | 10 (3.2) | 6.8% |
| North | 58 (5.3) | 21 (2.7) | 37 (11.8) | 13.3% |
| Nabatieh | 78 (7.1) | 22 (2.8) | 56 (17.8) | 7.9% |
| Skip this question | 17 (1.5) | 16 (2.0) | 1 (0.3) |  |
| **Religion** |  |  |  |  |
| Christian | 242 (21.9) | 199 (25.2) | 43 (13.7) | 32.4% |
| Druze | 355 (32.2) | 308 (39.0) | 47 (15.0) | 4.5% |
| Shi'a | 136 (12.3) | 46 (5.8) | 90 (28.7) | 31.0% |
| Sunni | 124 (11.2) | 64 (8.1) | 60 (19.1) | 31.9% |
| No religion | 65 (5.9) | 48 (6.1) | 17 (5.4) | NA |
| Other | 6 (0.5) | 3 (0.4) | 3 (1.0) | 0.3% |
| Skip this question | 175 (15.9) | 121 (15.3) | 54 (17.2) |  |
| **Highest Education Level** |  |  |  |  |
| Completed high school, technical school, or less | 200 (18.1) | 143 (18.1) | 57 (18.2) | 78.6% |
| Completed some college or more | 891 (80.9) | 637 (80.8) | 254 (80.9) | 21.4% |
| Skip this question | 11 (1.0) | 8 (1.0) | 3 (1.0) |  |
| **Employment** |  |  |  |  |
| Employed | 586 (51.5) | 412 (52.2) | 156 (49.7) | NA |
| Student | 179 (16.2) | 117 (14.8) | 62 (19.7) | NA |
| Unemployed, not seeking work | 145 (13.1) | 122 (15.5) | 23 (7.3) | NA |
| Unemployed, seeking work | 154 (14.0) | 101 (12.8) | 53 (16.9) | 33.0%^c^ |
| Skip this question | 57 (5.2) | 37 (4.7) | 20 (6.3) |  |
| **2019 annual income (LL)** |  |  |  |  |
| <1,000,000 | 122 (11.1) | 78 (9.9) | 44 (14.0) | Reliable data unavailable |
| 1,000,000-9,999,999 | 289 (26.2) | 199 (25.2) | 90 (28.7) |  |
| 10,000,000-19,999,999 | 99 (9.0) | 67 (8.5) | 32 (10.2) |  |
| 20,000,000-69,999,999 | 132 (12.0) | 100 (12.7) | 32 (10.2) |  |
| ≥70,000,000 | 61 (5.5) | 49 (6.2) | 12 (3.8) |  |
| Skip this question | 400 (36.3) | 296 (37.5) | 104 (33.1) |  |
| **Citizenship^a^** |  |  |  |  |
| Lebanon | 1038 (94.1) | 757 (95.9) | 281 (89.5) | Lebanon: 79.8% |
| Syria | 28 (2.5) | 6 (0.8) | 6 (1.9) | Not citizen of Lebanon: 20.2% |
| Palestine | 17 (1.5) | 12 (1.5) | 12 (3.8) |  |
| European or North American country | 40 (3.6) | 32 (4.1) | 32 (10.2) |  |
| Other country | 23 (2.1) | 16 (2.0) | 16 (5.1) |  |
| Skip this question | 12 (1.1) | 8 (1.0) | 8 (2.5) |  |
| Multiple countries | 53 (4.8) | 40 (5.1) | 40 (12.7) |  |
| **Refugee** |  |  |  |  |
| Yes | 56 (5.1) | 29 (3.7) | 27 (8.6) | 21.9%^c^ |
| No | 1015 (92.0) | 737 (93.4) | 278 (88.5) | 78.1%^c^ |
| Skip this question | 32 (2.9) | 23 (2.9) | 9 (2.9) |  |

The first column includes all participants aggregated. The second column describes those who completed the survey in the first round, prior to initiation of vaccination in Lebanon on 13 February 2021. The third column describes those who completed the survey in the second round, after initiation of vaccination in Lebanon. Because participants were not forced to answer all questions, this results in a different denominator for each question.

LL: Lebanese Lira

NA: Not available in data source

a) Participants could select multiple answers. Proportions were calculated using a denominator of all participants who selected an answer for the question.

b) Unless otherwise noted, estimates were obtained from government source that excluded refugees and used 4.84 million (2018) as total population.

c) Estimates were obtained from source that included refugees and used 6.86 million (2020) as total population.
