# Supplementary material for: Perceptions of, and Obstacles to, SARS-CoV-2 Vaccination Among Adults in Lebanon: Cross-sectional Online Survey
Source: JMIR Form Res. 2022 Dec 14;6(12):e36827. doi: 10.2196/36827 (PMC9762140; doi:10.2196/36827)
Supplement: Multimedia Appendix 5 [file formative_v6i12e36827_app5.docx]

**Table S2: Additional logistical considerations about vaccination**

| **Logistical Consideration** |  | **Participants, n (%)** |
| --- | --- | --- |
|  |  |  |
| **Among participants who did not intend to get vaccinated or were unsure about receiving a vaccine: Would a monetary incentive change your mind so that you take a vaccine?** |  |  |
| Yes |  | 10 (1.7%) |
|  |  |  |
| No |  | 589 (98.3%) |
|  |  |  |
|  |  |  |
| **Among all participants: What is the most of your own money that you would be willing to pay for a coronavirus vaccine?** |  |  |
| Zero LL |  | 201 (19.4%) |
|  |  |  |
| Between 1,000 LL and 10,000 LL |  | 86 (8.3%) |
|  |  |  |
| Between 11,000 LL and 20,000 LL |  | 120 (11.6%) |
|  |  |  |
| Between 21,000 LL and 50,000 LL |  | 147 (14.2%) |
|  |  |  |
| Between 51,000 LL and 75,000 LL |  | 64 (6.2%) |
|  |  |  |
| Between 76,000 LL and 100,000 LL |  | 119 (11.5%) |
|  |  |  |
| More than 100,000 LL |  | 113 (10.9%) |
|  |  |  |
| I will not get the coronavirus vaccine even if it is free |  | 186 (18.0%) |
|  |  |  |
|  |  |  |
| **Where would you prefer to get a coronavirus vaccine? Choose your top 3 preferred locations.^a,b^** |  |  |
| My doctor’s office |  | 257 (28.6%) |
|  |  |  |
| Any doctor’s office |  | 97 (10.8%) |
|  |  |  |
| A primary health center |  | 195 (21.7%) |
|  |  |  |
| A pharmacy |  | 140 (15.6%) |
|  |  |  |
| A hospital |  | 455 (50.6%) |
|  |  |  |
| A building close to my home that serves as a dedicated temporary vaccination clinic |  | 71 (7.9%) |
|  |  |  |
| A mobile van close to my home that serves as a dedicated temporary vaccination clinic |  | 41 (4.6%) |
|  |  |  |
| In my home, administered by a visiting certified vaccination provider |  | 106 (11.8%) |
|  |  |  |
| Other (Lebanese Red Cross, Military Primary Health Center, other health organization)^c^ |  | 13 (1.4%) |
|  |  |  |
|  |  |  |
| **Where do you receive most of your information and news about coronavirus? Choose your top 3 most common news sources.^a^** |  |  |
| Newspaper or magazine |  | 86 (7.8%) |
|  |  |  |
| Radio |  | 34 (3.1%) |
|  |  |  |
| Television |  | 721 (65.4%) |
|  |  |  |
| Social media (like Facebook, Twitter, YouTube, WhatsApp) |  | 678 (61.5%) |
|  |  |  |
| Internet, but not social media (like websites) |  | 611 (55.4%) |
|  |  |  |
| Talking to friends or family |  | 284 (25.7%) |
|  |  |  |
| Religious leaders |  | 6 (0.5%) |
|  |  |  |
|  |  |  |
| **Which news source do you trust the most for information and news about coronavirus? Choose your top 3 most trusted news sources.^a^** |  |  |
| Newspaper or magazine |  | 144 (13.8%) |
|  |  |  |
| Radio |  | 52 (5.0%) |
|  |  |  |
| Television |  | 627 (60.3%) |
|  |  |  |
| Social media (like Facebook, Twitter, YouTube, WhatsApp) |  | 299 (28.8%) |
|  |  |  |
| Internet, but not social media (like websites) |  | 639 (61.4%) |
|  |  |  |
| Talking to friends or family |  | 127 (12.2%) |
|  |  |  |
| Religious leaders |  | 12 (1.2%) |

This table shows responses to questions about logistical considerations about vaccination. For the analysis of each characteristic, we omitted participants who skipped the question, unless >10% of participants for that question skipped the question, in which case those who skipped the characteristic question were included in analysis.

^a^The survey allowed participants to choose multiple answers for this question; consequently, the sum of all subcategories does not equal the number of all participants who answered the question.

^b^Analysis for this question excludes those who skipped the question or who selected, “I will not get the coronavirus vaccine regardless of available locations.”

^c^If participants selected “Other,” they could write in their own response. The three responses in parenthesis (Lebanese Red Cross, Military Primary Health Center, other health organization) encompass the written-in responses.
